# ApproximateSecret Sharing in Field of Real Numbers

**DOI:** 10.3390/e27070769

**Published:** 2025-07-20

**Authors:** Jiaqi Wan, Ziyue Wang, Yongqiang Yu, Xuehu Yan

**Affiliations:** 1 College of Electronic Engineering, National University of Defense Technology, Hefei 230037, China; jiaqiwan2004@163.com (J.W.); 15211139646@163.com (Z.W.); publictiger@126.com (X.Y.); 2 Anhui Key Laboratory of Cyberspace Security Situation Awareness and Evaluation, Hefei 230037, China

**Keywords:** secret sharing, Shamir’s polynomial, common real numbers, type code, lossless, entropy

## Abstract

In the era of big data, the security of information encryption systems has garnered extensive attention, particularly in critical domains such as financial transactions and medical data management. While traditional Shamir’s Secret Sharing (SSS) ensures secure integer sharing through threshold cryptography, it exhibits inherent limitations when applied to floating-point domains and high-precision numerical scenarios. To address these issues, this paper proposes an innovative algorithm to optimize SSS via type-specific coding for real numbers. By categorizing real numbers into four types—rational numbers, special irrationals, common irrationals, and general irrationals—our approach achieves lossless transmission for rational numbers, special irrationals, and common irrationals, while enabling low-loss recovery for general irrationals. The scheme leverages a type-coding system to embed data category identifiers in polynomial coefficients, combined with Bernoulli-distributed random bit injection to enhance security. The experimental results validate its effectiveness in balancing precision and security across various real-number types.

## 1. Introduction

In recent years, with the rapid development of the economy, people’s demands for privacy protection, multi-party computing [1], IoT security [2], key management [3], and identity authentication [4,5] have grown exponentially. Since Shamir and Blakley first introduced the concept of secret sharing in 1979 [6,7], Shamir secret sharing (SSS) has gained widespread adoption in scenarios such as image transmission [8,9,10,11], edge computing [12], data management [13,14], and 5G networks [15], owing to its information-theoretic security and mathematical simplicity. However, the proliferation of diverse data types in these domains—ranging from floating-point weights in machine learning models [16] to decimal-based financial transactions [17] and irrational numbers in scientific computations—has exposed a critical limitation: SSS operates exclusively in finite domains like GF (p), restricting secrets and shares to integers.

For instance, in machine learning, neural network weights (typically floating-point numbers) [16] must be scaled to integers for sharing, requiring reverse quantization that may introduce errors. Financial transactions involving decimals [17] face similar challenges, with precision loss or increased computational complexity arising from integer conversion. Scientific computations, which often involve infinite recurring decimals and irrationals, are particularly vulnerable to significant errors due to truncation or approximation [18,19,20]. Meanwhile, applications in real-number fields [21,22] demand secure handling of non-integer data, highlighting the urgent need for SSS extensions beyond integer domains.

Existing solutions predominantly fall into two categories:

1. Floating-point arithmetic protocols [23,24], which encode different number types in finite fields and employ Boolean operations alongside SSS to ensure transmission security, though their effectiveness hinges on communication quality;
2. Decimal scaling methods, which convert secrets to integers for SSS, sacrificing support for irrational numbers altogether. Both approaches fail to address the fundamental challenge of representing irrational numbers precisely, leaving receivers unable to quantify accuracy degradation and introducing data loss in high-precision contexts.

Challenges: The precise representation of irrational numbers remains a computational hurdle, as truncating decimals or relying on approximations leads to irreversible information loss. Traditional methods, whether through encoding conversions or scaling, struggle to balance data integrity and transmission security, particularly in scenarios requiring high-fidelity secret reconstruction.

Contributions: Building on the core architecture of traditional SS, this paper introduces a novel type code system to enable real-number secret sharing. Real numbers are categorized into four types: rational numbers (finite decimals, fractions, infinite repeating decimals), general irrationals, special irrationals, and common irrationals, which are assigned unique type codes for backward compatibility and version-aware decoding. This framework ensures lossless transmission for rational numbers, special irrationals and common irrationals, while minimizing precision loss for general irrationals via bounded significant digit truncation. Security is reinforced through the following methods:

1. Bernoulli-distributed random bit injection, which guarantees uniqueness across different encoding instances of the same number, resisting statistical analysis-based brute-force attacks;
2. Type code obfuscation, which strategically distributes critical identifiers ) across non-adjacent encoding components, significantly increasing reverse-engineering difficulty;
3. Dynamic extension mechanisms for special irrational numbers, which enhance cryptographic resilience through position-variable encoding structures.

Inspiration from recent advancements in chaotic maps further enriches this work. For example, the 3D memristive cubic map with dual discrete memristors [25] demonstrates how hardware-implementable chaotic systems can generate high-quality random key streams for secure multi-party computation, while the DCT compression-based image privacy scheme [26] highlights the feasibility of integrating real-number processing with cryptographic primitives. These studies underscore the importance of hybrid architectures that handle mixed data types (integers, reals, irrationals) efficiently, informing our design of a versatile SSS framework for image encryption and beyond.

## 2. Preliminary

In this section, we introduced the basic definitions and preliminaries used in this paper, including SS number classification.

### 2.1. Shamir Secret Sharing

The Shamir secret-sharing scheme (SSS) [6,7] is a distributed cryptography protocol based on polynomial interpolation, which was proposed by Adi Shamir in 1979. The core idea is to divide the secret into multiple shares and ensure through a threshold mechanism that the secret can only be recovered when a sufficient number (threshold) of shares are collected. The core concept of the scheme is described below:

1. Secret (*r*): sensitive information that needs to be protected;
2. Share (SC): distribute the multiple data points generated after splitting the secret to different participants;
3. Threshold (*k*): the minimum number of shares required to restore the secrecy;
4. *n*: The total number of generated shares (n ≥ k);
5. Finite field, GF(p): all operations are carried out in the prime number field.

For the process of secret distribution, the first step is to set parameters. Select a large prime number *p*. The value of *p* should be as large as possible to avoid brute-force enumeration of the secret. Additionally, set the threshold *k* and the total number of shares *n*. Generate a polynomial:

(1) $$F\left(x\right)=r+a_{1}x+a_{2}x^{2}+...+a_{\left(k-1\right)}x^{\left(k-1\right)}\;\mathrm{mod}\;p$$

The constant term *r* refers to the secret that needs to be kept confidential. Each participant is assigned a unique non-zero identifier, $x_{i}$($x_{i}\in \left[1,n\right]$, and each $x_{i}$ is also different), which allows us to calculate the corresponding function values:

(2) $$SC_{i}=F\left(x_{i}\right)\;\mathrm{mod}\;p$$

Each share here represents ($x_{i}$,$SC_{i}$), Then, we distribute *n* shares.

When the receiver receives at least *k* shares, it can reconstruct the polynomial using Lagrange interpolation.

The SSS demonstrates several notable advantages. Primarily, it exhibits robust security properties, as an adversary possessing k−1 shares cannot obtain any information about the secret. Secondly, the scheme features a flexible threshold mechanism that supports arbitrary (k,n) threshold configurations, with the distinctive capability of adjusting thresholds without necessitating the regeneration of all shares. Furthermore, both the polynomial generation and Lagrange interpolation processes maintain a computational complexity of O(k), resulting in low computational overhead for secret recovery. This efficiency renders the scheme particularly suitable for large-scale distribution scenarios.

However, the scheme is not without limitations.A critical vulnerability arises from potential share corruption during transmission, which can be caused by either adversarial actions (e.g., attackers forging shares during distribution) or unintentional errors introduced by the distributor. Such corruptions can lead to erroneous secret reconstruction. Additionally, when dealing with lower-degree polynomials (e.g., *k* = 2), the scheme may exhibit structural weaknesses that could compromise its security robustness.

### 2.2. Number Classification

We classify real numbers into four categories: rational numbers, general irrational numbers, special irrational numbers, and common irrational numbers.

1. Rational number (Q): A number of the form $\frac{a}{b}$, where a,b∈Z and b≠0. *a* is the numerator.
2. Special irrational number (${\mathrm{CrQ}}_{s}$): An irrational number that can be simplified to a rational form after raising to a power. It is specifically expressible as $\sqrt[{I}]{\frac{a}{b}}$, where
  $$a,b\in Z,\;b\neq 0,\;I\in N^{+}\;\left(I\geq 2\right)$$
  If *I* is even, $\frac{a}{b}\geq 0$ must hold.
3. Common irrational number (${\mathrm{CrQ}}_{p}$): Numbers of the form $\frac{\pi }{v}$ or $\frac{e}{v}$, where $v\in N^{*}$ (e.g., $\frac{\pi }{2}$, $\frac{e}{5}$). Note: Only strictly $\frac{\pi }{v}$ or $\frac{e}{v}$ forms are recognized as common irrational numbers. Numbers like $e^{3}$ or $\pi^{2}$ or $\pi^{e}$ are classed as general irrational numbers.
4. General irrational number (${\mathrm{CrQ}}_{G}$): Numbers satisfying r∈R and $r\notin {\mathrm{CrQ}}_{p}\cup Q\cup {\mathrm{CrQ}}_{s}$. Examples include
  $$2\ln2,\;e^{3}$$

## 3. The Proposed Scheme

This section is organized into three sections. The first section provides an overview of our proposed scheme. The second section delves into the design approach and its underlying principles. The final section offers a general discussion of the scheme’s characteristics and implications.

### 3.1. Overview

**Figure 1:**
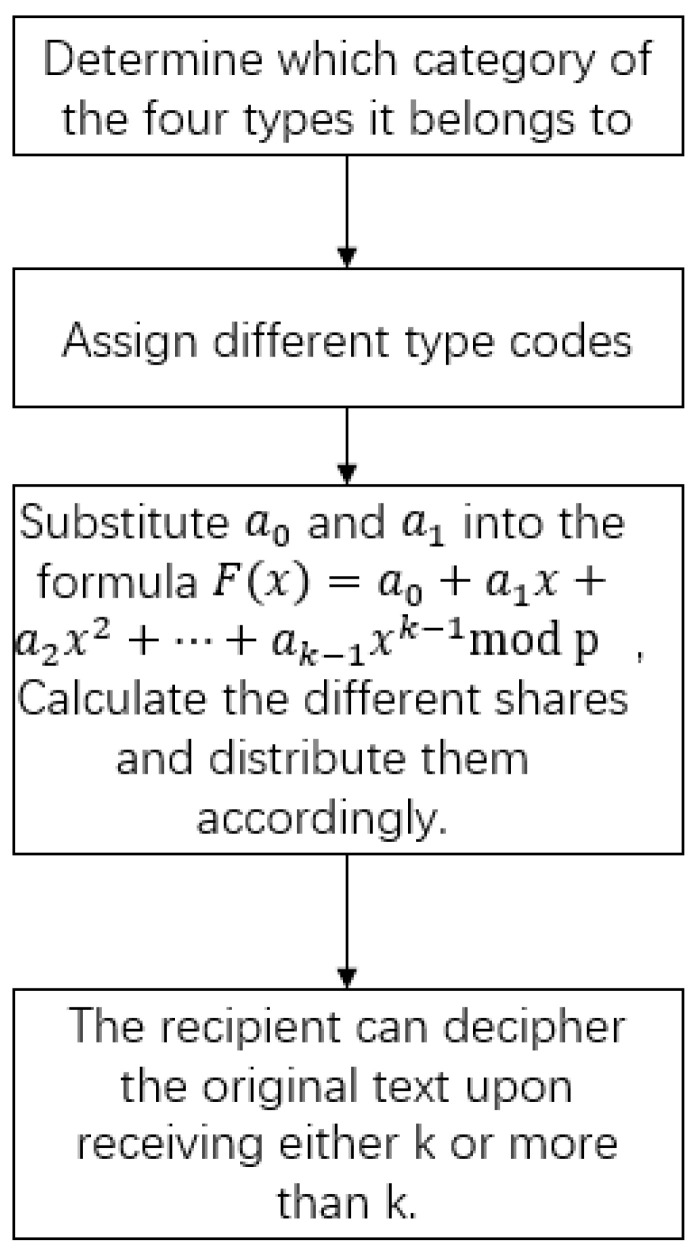
Algorithm flowchart.

Existing schemes are often limited to a certain bit range for secret sharing, discarding any fractional parts beyond that range. The receiver cannot determine whether the received data has lost part of its precision. To address this issue, we leverage the $a_{1}$ component of the SSS to embed specific information reflecting its type, ensuring security by incorporating substantial random bits into $a_{1}$. Due to the complexity of real numbers, as mentioned in the previous text, we classify them into four categories: rational numbers, general irrational numbers, common irrational numbers, and special irrational numbers. We assign specific type codes to each category so that the receiver can fully obtain the original information. The basic algorithm approach is as follows Figure 1:

The symbol explanations in this scheme are as follows Table 1:

### 3.2. Algorithm Design

The algorithm can be divided into three main steps: real number standardization, type code length determination, and encoding structure specification.

1. **Real number standardization**
  If $r\in GrQ_{G}$, its scientific notation representation is given by
  $$r=a\times 10^{\lambda },\;\mathrm{where}\;1\leq \left|a\right|<10\;\mathrm{and}\;\lambda \in Z.$$
  We extract the first eight significant digits from *a* and perform rounding to obtain α, satisfying
  $$\alpha =\mathrm{Round}\left(a,8\right)=a+O\left(10^{-8}\right),$$
  where Round(a,8) denotes rounding *a* to eight significant digits. Then, the preprocessed form of *r* is
  $$r=\alpha \times 10^{\lambda }.$$
  Perform standardization transformation on secret r∈R:
  $$r\mapsto \left\{\begin{array}{cc} \frac{a}{b},\;a,b\in Z,\;b\neq 0 & \mathrm{if}\;r\in Q\\ \alpha \times 10^{\lambda } & \mathrm{if}\;\lambda \in N,\;\mathrm{and}\;r\in {\mathrm{CrQ}}_{G}\\ \frac{\pi }{v}\;\mathrm{or}\;\frac{e}{v},\;v\in Z,\;v\neq 0 & \mathrm{if}\;r\in {\mathrm{CrQ}}_{p}\\ \sqrt[{I}]{\frac{a}{b}},\;a,b,I\in Z,\;b,I\neq 0 & \mathrm{if}\;r\in {\mathrm{CrQ}}_{s} \end{array}\right.$$
2. **Type code length determination**
  The type code length ℓ(T) follows
  $$\ell \left(T\right)=\left\{\begin{array}{cc} 8 & \mathrm{if}\;r\in Q\\ 16 & \mathrm{if}\;r\in {\mathrm{CrQ}}_{p}\\ 4\cdot \ell (W) & (\mathrm{dynamic}\;\mathrm{extension}\;\mathrm{mechanism})\;\mathrm{if}\;r\in {\mathrm{CrQ}}_{s}or\;r\;\in {\mathrm{CrQ}}_{G} \end{array}\right.$$
  For special irrational numbers (${\mathrm{CrQ}}_{s}$), ℓ(T) is determined by a dynamic extension mechanism. Let the bit-length of radical *I* be
  $$\ell \left(W\right)=2^{\left\lceil \log_{2}\left(\mathrm{len}\left(\mathrm{bin}\left(I\right)\right)\right)\right\rceil }$$
  For general irrational numbers, the determination of ℓ(T) also adopts a dynamic expansion mechanism.
  The dynamic expansion mechanism automatically adjusts the length based on the binary representation length of λ.
  The code length parameters for different types are shown in Table 2:
  **Table 2:** Code length determination rules.

Type	ℓ(T)	ℓ(W)	ℓ(Random)	Code Length Determination Rule
Rational Number	8	0	4	ℓ(T)=8
General Irrational	4·ℓ(W)	$2^{\left\lceil \log_{2}\ell \left(\mathrm{bin}\left(\lambda \right)\right)\right\rceil }$	3·ℓ(W)−4	Dynamic expansion mechanism
Common Irrational	16	1	11	ℓ(T)=16
Special Irrational	4·ℓ(W)	$2^{\left\lceil \log_{2}\left(\ell \left(\mathrm{bin}\left(n\right)\right)\right)\right\rceil }$	3·ℓ(W)−4	Dynamic expansion mechanism
3. **Encoding Structure Definition**
  The type code $T=(t_{1}t_{2}\ldots t_{n})$ follows a strict binary concatenation scheme:
  $$T=t_{1}t_{2}\|t_{3}\|W\|\mathrm{Random}\|s$$
  with components defined as
  (a) *Data type code* ($t_{1}t_{2}$):
    $$\left(t_{1}t_{2}\right)=\left\{\begin{array}{cc} 00 & r\in Q\\ 01 & r\in {\mathrm{CrQ}}_{s}\\ 10 & r\in {\mathrm{CrQ}}_{G}\\ 11 & r\in {\mathrm{CrQ}}_{p} \end{array}\right.$$
  (b) *Component flag* ($t_{3}$):
    $$t_{3}=\left\{\begin{array}{cc} 0 & S(r)=a\\ 1 & S(r)=b \end{array}\right.\;\mathrm{where}\;S\left(r\right)=\left\{\begin{array}{cc} a & \left(\mathrm{numerator}\right)\\ b & \left(\mathrm{denominator}\right) \end{array}\right.$$
    Among them, if the secret is a general irrational number, then this bit is transformed into a reserved sign bit, which is used to mark the sign of the reserved bit:
    $$t_{3}=\left\{\begin{array}{cc} 0 & \mathrm{if}\;\lambda \geq \;0\\ 1 & \mathrm{if}\;\lambda \leq 0 \end{array}\right.$$
  (c) *Reserved Field* (*W*):
    $$W=\left\{\begin{array}{cc} 1 & \mathrm{if}\;r=\pi \\ 0 & \mathrm{if}\;r=e\\ \underset{\ell (T)-\ell (\mathrm{bin}(n))}{\underset{︸}{00\ldots 0}}∥\mathrm{bin}\left(n\right) & \mathrm{if}\;r\in {\mathrm{CrQ}}_{s}\mathrm{or}\;r\in {\mathrm{CrQ}}_{G} \end{array}\right.$$
    where ℓ(I)=len(bin(I)) and $\ell \left(W\right)=2^{\left\lceil \log_{2}\ell \left(I\right)\right\rceil }$
  (d) *Random Bits* (Random):
    $$\mathrm{Bernoulli}\left(0.5\right),\;\ell \left(\mathrm{Random}\right)=\left\{\begin{array}{cc} 4 & r\in Q\\ 11 & r\in {\mathrm{CrQ}}_{p}\\ \ell (T)-\ell (D)-\ell (W)-1 & r\in {\mathrm{CrQ}}_{s}\cup {\mathrm{CrQ}}_{G} \end{array}\right.\;\;\;\;\;\;\;\;\;\;\;\;\;\;\;\;\;\;$$
  (e) *Sign Bit* (*s*):
    $$s=\left\{\begin{array}{cc} 0 & r\geq \;0\\ 1 & r<0 \end{array}\right.$$
  The specific parameter settings are shown in the Table 3.
  **Table 3:** Encoding structure parameters.

Field	Length	Value Space	Description
$t_{1}t_{2}$	2 bits	00-11	Data type
$t_{3}$	1 bit	0-1	Marker bit or Reserved sign bit
*W*	Variable	Binary	Reserved bit
Random	Variable	Bernoulli (0.5)	Random bit
*s*	1 bit	0-1	Positive and negative identification bit
4. **Case Studies**
  (a) *Special irrational number example*: $\sqrt[{20000}]{\frac{2}{1}}$
    - Data type: $t_{1}t_{2}=01$ (special irrational)
    - Maker bit: $t_{3}=0$ (numerator encoding for 2)
    - Reserved field (*W*):
      I=20000⇒bin(I)=100111000100000,ℓ(W)=16ℓ(T)=64
      $$W=\underset{1-\mathrm{bit}\;\mathrm{pad}}{\underset{︸}{0}}\|100111000100000$$
    - Final type code structure for numerator: 0100100111000100000XX⋯Xs
  (b) *Common irrational number example*: $\frac{\pi }{4}$
    - Type code: $t_{1}t_{2}=11$ (common irrational)
    - Component flag: $t_{3}=0$ (numerator π)
    - Reserved field (W=1 for π)
    - Final type code for numerator: $\underset{\mathrm{type}}{\underset{︸}{11}}\;\underset{\mathrm{marker}}{\underset{︸}{0}}\;\underset{W}{\underset{︸}{1}}\;\underset{\mathrm{Random}}{\underset{︸}{\mathrm{XXXXXXXXXXX}}}\;\underset{\mathrm{sign}}{\underset{︸}{0}}$
5. **Secret reconstruction**
  When *k* valid shares are received, reconstruct the polynomial using Lagrangian interpolation:
  (a) Define basis polynomials:
    $$L_{i}\left(x\right)=\prod_{\begin{array}{c} 1\leq j\leq k\\ j\neq i \end{array}}\frac{x-x_{j}}{x_{i}-x_{j}}\;\mathrm{mod}\;p$$
    satisfying
    $$L_{i}\left(x_{j}\right)=\left\{\begin{array}{cc} 1 & \mathrm{if}\;i=j\\ 0 & \mathrm{if}\;i\neq j \end{array}\right.$$
  (b) Reconstruct the polynomial:
    $$F\left(x\right)=\sum_{i=1}^{k}y_{i}\cdot L_{i}\left(x\right)\;\mathrm{mod}\;p$$
  (c) Recover secret coefficients:
    $$\begin{array}{cc} a_{0} & =F(0)\\ a_{1} & =\mathrm{Solve}\;\mathrm{from}\;F\left(x\right)\;\mathrm{using}\;a_{0} \end{array}$$
6. **Theoretical error bounds and error distribution analysis**
  For general irrational numbers, analyze the error bounds and error distribution when truncating to eight decimal places.
  (a) The theoretical error bound: Suppose *x* is an irrational number, its true value is *x*, and the value after truncation to eight decimal places is $x^{\prime }$. Then the truncation error ϵ can be expressed as
    (3) $$\epsilon =|x-x^{\prime }|$$
    Since it is truncated to eight decimal places, the maximum possible error occurs at the ninth decimal place. Therefore, the theoretical error bound can be expressed as
    (4) $$\epsilon \leq 0.5\times 10^{-8}$$
    This is because the maximum error occurs when the ninth digit is five (in the case of rounded boundaries), at which point the error is $0.5\times 10^{-8}$.
  (b) Hypothesis of error distribution: Suppose the truncation error ϵ is uniformly distributed within its theoretical error range. That is,
    (5) $$\epsilon ∼Uniform(0,0.5\times 10^{-8})$$
    This means that the error ϵ is uniformly distributed over the interval $\left[0,\;0.5\times 10^{-8}\right]$. This assumption is based on the following considerations:
    i Randomness: The decimal part of an irrational number is random, so the truncation error can be regarded as random.
    ii Uniform distribution assumption: Since the decimal parts of irrational numbers exhibit no regularity, it is reasonable to assume that truncation errors will arise within the error bounds predicted by uniform distribution theory.
  (c) Experimental verification. The experimental results mentioned in the paper show that the actual error is usually on the order of $10^{-8}$, which is consistent with the theoretical error bound and error distribution assumptions. Specifically,
    i Maximum error: The maximum error observed in the experiment is close to $0.5\times 10^{-8}$, which is consistent with the theoretical error bound.
    ii Average error: As the error is uniformly distributed within $\left[0,\;0.5\times 10^{-8}\right]$, the average error is approximately $0.25\times 10^{-8}$.
    Through theoretical analysis and experimental verification, we can draw the following conclusions:
    i Theoretical error bound: For an ordinary irrational number truncated to eight decimal places, its theoretical error bound is $0.5\times 10^{-8}$.
    ii The error distribution hypothesis: There will be truncation errors in $\left[0,\;0.5\times 10^{-8}\right]$ uniform distribution. This is a reasonable and acceptable assumption, and this was also confirmed in the experiment.

### 3.3. General Analysis

#### 3.3.1. Rational and General Irrational Numbers: General Proof

Assuming the secret to be transmitted is r∈Q, perform standardization by expressing *r* as a/b. For numerator *a*, assign data type 000XXXXs, and for denominator *b*, assign data type 001XXXX0 according to previous algorithms. Construct two independent polynomials for the SS scheme:

$$F_{a}\left(x\right)=a_{0}^{\left(a\right)}+a_{1}^{\left(a\right)}x+a_{2}^{\left(a\right)}x^{2}+\cdots +a_{k-1}^{\left(a\right)}x^{k-1}\;\mathrm{mod}\;p$$

$$F_{b}\left(x\right)=a_{0}^{\left(b\right)}+a_{1}^{\left(b\right)}x+a_{2}^{\left(b\right)}x^{2}+\cdots +a_{k-1}^{\left(b\right)}x^{k-1}\;\mathrm{mod}\;p$$

where $a_{0}^{\left(a\right)}=a$, $a_{0}^{\left(b\right)}=b$, $a_{1}^{\left(a\right)}=T_{a}$, and $a_{1}^{\left(b\right)}=T_{b}$. Generate *n* shares for each of *a* and *b*:

$${\left\{\left(x_{i},F_{a}\left(x_{i}\right)\right)\right\}}_{i=1}^{n}\;\mathrm{and}\;{\left\{\left(x_{i},F_{b}\left(x_{i}\right)\right)\right\}}_{i=1}^{n}$$

If the receiver obtains *k* or more shares, they can reconstruct $a_{0}$ and $a_{1}$, thereby recovering the original numerator *a*. Similarly, the denominator *b* can be recovered, reconstructing the original number a/b. This demonstrates the generality of the scheme. For regular irrational numbers, simply take eight decimal places and change the type code to 00. Algorithm 1 presents the secret-sharing scheme for rational numbers.
**Algorithm 1** Secret-sharing encryption algorithm for rational numbers**Require:** r∈Q **Ensure:** $F(x_{i})$ where $x_{i}\in \left[1,n\right]$
  1: Step 1: Convert *r* to a/b form, where $T_{a}=000\mathrm{XXXXs}$ and $T_{b}=001\mathrm{XXXX}0$
  2: Step 2: For $T_{a}$, set $a_{1}=T_{a}$ and $a_{0}=a$. Substitute this into the polynomial$$F\left(x\right)=a_{0}+a_{1}x+a_{2}x^{2}+\cdots +a_{k-1}x^{k-1}\;\mathrm{mod}\;p$$
  3: Step 3: Output $F(x_{i})$ where $x_{i}\in \left[1,n\right]$


Note: Ensure all mathematical operations are performed in the finite field modulo *p*, where *p* is a large prime number greater than all possible coefficients.

#### 3.3.2. General Proof for Special Irrational Numbers

Assume the special irrational number to be transmitted is $\sqrt[{I}]{a/b}$. First compute W=bin(I). Following the prefix allocation principle described previously, the type codes are 010WXX…Xs and 011WXX…Xs for the numerator and denominator, respectively.

For the numerator’s polynomial construction,

$$F\left(x\right)=a_{0}+a_{1}x+a_{2}x^{2}+\cdots +a_{k-1}x^{k-1}\;\mathrm{mod}\;p$$

where $a_{0}=a$ (numerator value) and $a_{1}=010\mathrm{WXX}$…X. Select *n* shares $x_{1},x_{2},\ldots ,x_{n}$, compute corresponding $F(x_{i})$ values, then distribute all shares. If receivers obtain more than *k* shares, they can reconstruct $a_{0}$ and $a_{1}$.

The receiver recognizes special irrational numbers through the 01 prefix and determines numerator/denominator from the third bit. The data type length reveals ℓ(W). By removing the leading 0, *I* is recovered for radical reconstruction. The denominator follows the same procedure.The entire algorithm process is as shown in the Algorithm 2.
**Algorithm 2** Secret-sharing encryption algorithm for special irrational numbers**Require:** $\sqrt[{I}]{a/b}\in CrQ_{s}$ **Ensure:** $F(x_{i})$ where $x_{i}\in \left[1,n\right]$
  1: Step 1: I→bin(I)→W
  2: Step 2: Assign type codes:
  3: $T_{a}\to 010\mathrm{WXXX}$⋯Xs (numerator),
  4: $T_{b}\to 011\mathrm{WXX}$⋯X0 (denominator)
  5: Step 3: For numerator *a*, set coefficients:
  6: $a_{1}\leftarrow T_{a},\;a_{0}\leftarrow r$. Construct polynomial:$$F\left(x\right)=a_{0}+a_{1}x+a_{2}x^{2}+\cdots +a_{k-1}x^{k-1}\;\mathrm{mod}\;p$$
  7: Step 4: Output shares $F(x_{i})$, $\forall x_{i}\in \left[1,n\right]$


**Key features**:

- Type code structure embeds radical degree *I* in binary form (W).
- Header bits 01 identify special irrationals.
- Third-bit differentiation: 0 for numerator, 1 for denominator.

Note: The denominator polynomial construction follows the same pattern with $T_{b}$ and $b_{0}=\mathrm{denominator}\;\mathrm{value}$. All arithmetic operations must preserve the *W*-bit radical specification field during reconstruction.

#### 3.3.3. A General Analysis of the Rational Nature of Common Irrationals

To transmit the commonly used irrational number $\frac{m}{v}$, where *m* is one of the common irrational numbers π, *e*, we assign a type code to both the numerator and the denominator. The type code for the numerator is encoded as 110WXXXXXXXXXXXs (where the reserved bits are determined by the specific irrational number being transmitted; if it is π, the reserved bit is 0, and if it is *e*, the reserved bit is 1). The type code for the denominator depends on its specific type.

For the numerator, we select $x_{i}$, i∈[1,n] as a set of values. We evaluate as the Algorithm 3, resulting in $F(x_{i})$, i∈[1,n]. After obtaining all these evaluations, we distribute them. If the receiver obtains *k* or more evaluations, they can solve for the coefficients $a_{1}$. By examining the prefix 110 *W* and the sign bits, we can determine the numerator *m*. Similarly, we can decode the denominator *v*, thereby obtaining the original number $\frac{m}{v}$.
**Algorithm 3** The secret-sharing encryption algorithm for common irrationals**Input:** $\frac{m}{v},v\in CrQ_{p},v\in R$; **Output:** $F(x_{i})$, $x_{i}\in \left[i,n\right]$;
 Step 1: $T_{m}=110WXXX...Xs$,$T_{v}=001XXXXs$;
 Step 2: For *v*, set $a_{1}=T_{v}$ and $a_{0}=v$, substitute into $F\left(x\right)=a_{0}+a_{1}x+a_{2}x^{2}+a_{3}x^{3}+\cdots +a_{k-1}x^{k-1}\;\mathrm{mod}\;p$;
 Step 3: Output $F(x_{i})$, $x_{i}\in \left[1,n\right].$


### 3.4. The General Analysis of the Rational Nature of General Irrationals

Assume that the general irrational number to be transmitted is in the form $\alpha \times 10^{\lambda }$. It is known that the type code for the numerator is 10, and the reserved bits are sign(λ)||00..0||bin(λ). If λ is positive, the sign bit of the reserved field is 0; if negative, it is 1. The sign bit is determined by the sign of α, following the same principle as above. After transmission, if the receiver obtains *k* or more shares, they can decode the type code to identify the number as a general irrational number, and can then decode the values of α and λ to reconstruct the original secret *r*.The entire algorithm process is as shown in the Algorithm 4.
**Algorithm 4** Secret-sharing for general irrational numbers**Require:** $\alpha \times 10^{\lambda }$, $r\in {\mathrm{CrQ}}_{G}$ **Ensure:** $F(x_{i})$, $x_{i}\in \left[1,n\right]$
  1: Step 1: T←100WXX…Xs if λ≥0
  2: Step 2: Set $a_{1}=T$, $a_{0}=\alpha$, compute:
  3: $F\left(x\right)=a_{0}+a_{1}x+a_{2}x^{2}+\cdots +a_{k-1}x^{k-1}\;\mathrm{mod}\;p$
  4: Step 3: **return** $F\left(x_{i}\right)\;\forall x_{i}\in \left[1,n\right]$


#### 3.4.1. Security Analysis

In the case of the SSS, while attempts have been made to attack this, thus far, no efficient solution been found [1,27,28].

Initially, the receiver obtains *m* shares (where m≤k), corresponding to *m* equations with *k* unknowns, forming an underdetermined system. To ensure security, *k* and *n* should be as large as possible [29]. The entropy of each share determines its safety. If the entropy of the share is high enough, the original secret cannot be deduced even if the attacker gains part of the share.

The solution space has a dimension of k−m (number of free variables), with infinitely many polynomials satisfying the conditions. For any guessed $a_{0}$, we can construct a polynomial $F^{\prime }(x)$ such that $F^{\prime }\left(x\right)=y_{i}$ and $F^{\prime }\left(0\right)=a_{0}$. Therefore, all possible $a_{0}$ are equally likely candidates and cannot distinguish the true secret.

Thus, fewer than *k* shares result in an infinitude of solutions that prevent any information about the secret *r* from being revealed to an attacker (information-theoretic security). This is the core security guarantee of the SSS, which does not rely on computational assumptions but is based on the mathematical properties of polynomial interpolation [29].

For counterfeit attacks targeting data type codes, our scheme embeds the data type code as a secret within polynomials. If an attacker attempts to forge the type code, they must modify the shares $\left(x_{i},y_{i}\right)$. However, such modifications inevitably alter the decrypted secret value, thereby contradicting the attacker’s objective of misleading the receiver. The receiver must validate received shares and discard those that produce evidently erroneous decrypted data to mitigate such attacks.

Regarding random-bit enumeration attacks, while a smaller random-bit space theoretically allows attackers to rapidly enumerate and compromise secrets, our scheme explicitly specifies that the parameter $a_{1}$ must be at least 8 bits in length. Additionally, the random padding bits are independent of the type code, rendering such attacks practically infeasible in real-world deployments.

#### 3.4.2. Analysis of Algorithm Time and Space Complexity

Regarding time complexity, our algorithm relies on the Shamir encryption scheme. Consequently, the algorithm’s time complexity may be influenced by functions related to data type classification and type code generation. Based on our defined function for classifying data types into rational numbers, general irrational numbers, special irrational numbers, and commonly used irrational numbers, this function operates in constant time, O(1), with negligible impact on the overall algorithm’s time complexity. The function responsible for generating type codes for encrypted data constructs a two-digit type code along with a sign indicator, followed by additional bits based on the specific data type. This type code generation function also operates in constant time, O(1), with minimal impact on the overall time complexity. The algorithm’s time complexity is therefore equivalent to that of the Shamir encryption scheme, which is $O(n^{2})$.

Regarding space complexity, the analysis process is similar to that of time complexity. We now analyze the space complexity for the functions responsible for data type classification and type code generation. For the function responsible for classifying data types into different categories (rational numbers, general irrational numbers, special irrational numbers, and commonly used irrational numbers), no additional storage of data is required. Thus, its space complexity is O(1). For the type code generation function, the space complexity is determined by the fixed amount of space needed to store the returned type code. Since the size of the type code is predetermined and does not depend on the input, this function also has a space complexity of O(1). Both functions have negligible impacts on the overall space complexity of the algorithm. Therefore, the algorithm’s space complexity is equivalent to that of the Shamir encryption scheme, which is $O(k\log_{2}k)$.

#### 3.4.3. The Trade-Offs Between Algorithm Parameters

Next, we will discuss the trade-offs between the parameters k,p and the thresholds n,k:

1. The parameters *k* (threshold):
  (a) Security: the greater the *k* value, the higher the security of the system. Because attackers need to obtain a larger share to restore the secret, the risk of secret leakage is reduced. For example, when *k* is 4, attackers need to obtain at least four shares to recover the secret. Compared with when *k* is 2, the security has been significantly improved.
  (b) Efficiency: The larger the *k* value, the greater the computational complexity and communication overhead of the secret segmentation and recovery process. In the secret split phase, the polynomial and share computing time complexity are O(k), whereas in the secret recovery phase, the Lagrange interpolation method of time complexity is $O(k^{2})$. Meanwhile, more shares need to be stored and transmitted, which increases the space and communication costs.
  (c) Flexibility: A larger *k* value can offer higher fault tolerance. Even if some shares are lost or damaged, as long as the remaining share quantity reaches *k*, the secret can still be restored. For example, in a scenario with 10 participants and a threshold of five, even if four shares are lost, the secret can still be recovered.
2. Parameter *p* (prime numbers in a finite field):
  (a) Security: Choosing a larger prime number *p* can enhance the security of the system, as a larger finite field increases the difficulty of brute-force cracking secrets. For example, when choosing a 256-bit prime number *p*, compared with choosing a 64-bit prime number *p*, under the same conditions, the time and computing resources required for brute-force cracking will increase significantly.
  (b) Efficiency: A larger p-value will lead to an increase in computational and storage overhead. Operations are carried out in the finite field GF(p), including the generation of polynomials, the calculation of shares, and Lagrange interpolation, etc. The time complexity is proportional to the magnitude of *p*. Meanwhile, the storage space required for the storage share and polynomial coefficients will also increase as *p* increases.
3. Parameter *n* (total share quantity):
  (a) Fault tolerance: The larger the value of *n*, the stronger the fault tolerance of the system. Because there are more shares to choose from, even if some shares are lost or damaged, as long as the remaining share quantity reaches *k*, the secret can still be restored. For example, in a scenario with 20 shares and a threshold of five, even if 15 shares are lost, the secret can still be recovered.
  (b) Efficiency: The larger the value of *n*, the greater the overhead of secret segmentation and storage. More shares need to be generated and stored, which increases the space and communication costs. Meanwhile, in the secret recovery stage, *k* shares need to be selected from more shares for interpolation operation, which may increase the computational overhead.
  (c) Flexibility: A larger *n* value can offer greater flexibility. Different numbers of participants can be selected based on actual needs, and the allocation and recovery process of shares can be dynamically adjusted according to the availability of participants.

In practical applications, parameters k,p and *n* need to be reasonably selected based on specific security requirements, efficiency requirements, fault tolerance capabilities, etc., in order to achieve the best balance between performance and security. For example, in financial transaction scenarios with high security requirements, larger *k* values and *p* values can be selected to ensure the security of secrets. In Internet of Things (IoT) devices with high efficiency requirements, smaller *k* and *p* values can be selected to reduce computing and communication overhead.

#### 3.4.4. Performance Analysis

Our scheme can realize lossless transmission in rational numbers, common irrational numbers, and special irrational numbers. When the receiver receives the original ciphertext and type code, it can determine the exact type of the original integer received and then restore it. For the general irrational numbers, the secret of eight decimal places can be accurately restored so as to minimize the loss of the original secret.

Our scheme not only minimizes losses during secret recovery but also maintains a high level of security. By incorporating more than half of the random digits in the type code method, we effectively prevent brute-force attacks by adversaries.

## 4. Experiments and Comparisons

In this section, the experimental data and results demonstrate that the proposed scheme achieves its intended performance by enabling secret sharing of different input data formats. The experiments are conducted using four types of raw ciphertexts and aim to validate the proposed scheme through experimental content including the SS of rational numbers, the SS of general irrational numbers, the SS of common irrational numbers, the SS of special irrational numbers, error rate comparisons, and error analysis.

The experiments were conducted on a Windows 11 system. The implementation was carried out in Python 3.12, leveraging libraries such as openpyxl for data handling. For real numbers, the study primarily distinguished between rational and irrational numbers. Irrational numbers were further categorized into general irrational numbers, common irrational numbers (such as π and *e*), and special irrational numbers with the aim of enabling lossless transmission. Based on these categorizations, experiments were conducted across the four categories to verify the correctness of the original number generation for both the sender and receiver. The evaluation focused not only on whether the original number could be accurately generated but also on comparing our proposed scheme with existing schemes to highlight its advantages.

### 4.1. The SS of Rational Numbers

**Figure 2:**
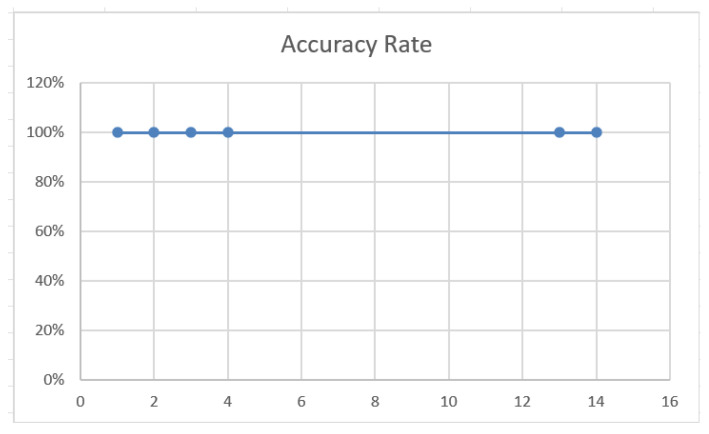
The accuracy of the rational numbers experiment.

The experimental data range from −10,000 to 10,000, including 1300 rational numbers (both positive and negative), integers, fractions, and decimals with up to twelve decimal places. For example, the number −1741.6714588027 is selected to illustrate the encryption process for a general negative fraction. First, we convert the number into its fractional form: −17,416,714,588,027/10,000,000,000. The numerator and denominator are then encoded separately according to their type codes. Using the SSS, we split the numerator (*a*_0_ = 17,416,714,588,027) and the denominator (*a*_1_ = 10,000,000,000), where *k* = 4 and *n* = 5. We perform the operation as described in Algorithm 1, and thus obtain the secret. Below are the results of this experiment on the 1300 data points as shown in Table 4, Figure 2.

Table 4 summarizes the number of data types along with their corresponding error rates. Figure 2 presents the accuracy changes under different digitization conditions. Based on these observations, we can also derive the following conclusions:

1. For rational numbers, our protocol scheme is error free within a fixed range of numbers, where the length of the number (the maximum number of digits in the numerator or denominator expressed as a fraction) does not affect the error rate. This is because, within a limited number of digits that can be represented by a computer without considering rational number overflow, there is no error inherent in the experimental transmission process.
2. The protocol introduces randomness during the generation of shares, which means that even if two shares correspond to the same secret value, they will appear completely different. This significantly increases the security complexity and ensures that at least *k* shares are required to reconstruct the original secret. Even if an attacker obtains fewer than *k* shares, no information about the original secret can be revealed.
3. The SSS is adopted. By adjusting *k* values, different levels of security protection can be achieved. Specifically, in the example, parameters are set as k=4, n=5, meaning that the original secret is split into five shares (*n* = 5), and any combination of four shares is sufficient to reconstruct the original secret. This mechanism allows for flexible threshold settings, enhancing both the security and flexibility of data transmission.

### 4.2. The SS of General Irrational Numbers

For general irrational numbers, we selected a total of 300 common irrational numbers, including complex radicals such as $\sqrt[{3}]{4}+1$ and $2\sqrt{2\pi }$, and those with extremely small values in scientific notation (e.g., Planck’s constant $h=6.62607015\times 10^{-34}$). These numbers have the characteristic that they cannot be precisely represented during transmission and inherently contain errors. In our specific experiment, we retained eight significant digits for sharing. For instance, let us Take $4\sqrt[{3}]{\pi }$ and Planck’s constant *h* as examples.

Approximating $4\sqrt[{3}]{\pi }$ with eight decimal places resulted in the value 5.85836755, which corresponded to the fraction 117,167,351/20,000,000. We performed the operation as described in Algorithm 1, and thus obtained the secret.

Planck’s constant *h* (6.62607015 × 10^−34^) is truncated to 6.6260702 × 10^−34^ (retaining 8 significant digits from the first non-zero digit). In the reserved bits of the protocol, 8 bits are retained to fill in the binary of 34 (00100010), indicating that there are 34 zeros before the significant digits of this data, and then random bits are filled in to supplement the protocol. The remaining data retains eight significant digits of 6.6260702, which is converted into the fraction 66,260,702/10,000,000, and encrypted according to the operations of Algorithm 1 to obtain the secret information.

The theoretical error bound for 8 significant digits is derived as

(6) $$\epsilon \leq 0.5\times 10^{-(m-8)}$$

where m is the position of the first non-zero digit. For *h* (m=34), the error bound is $0.5\times 10^{-26}$, while for $4\sqrt[{3}]{\pi }$ (m=1), it is $0.5\times 10^{-8}$. This mechanism ensures consistent precision across different orders of magnitude.

Experimental results for 300 test cases (including 50 data points with extremely small values such as physical constants and quantum parameters) show

1. Maximum error: $4.9\times 10^{-9}$ for $4\sqrt[{3}]{\pi }$, $3.1\times 10^{-27}$ for h, aligning with theoretical bounds;
2. Average error: $2.5\times 10^{-9}$ for large-magnitude irrationals, $1.7\times 10^{-27}$ for data with extremely small values, confirming uniform distribution within $\left[0\;,0.5\times 10^{-(m-8)}\right]$.

Through the above data, we can draw the following conclusion:

1. Eight significant digits truncation reduces precision loss by 2–3 orders of magnitude for data with extremely small values compared to decimal truncation;
2. The error rate remains stable at $10^{-8}$ to $10^{-27}$ across data scales, demonstrating universality;
3. For irrational numbers in general, the protocol achieves a balance between security and effectiveness. In experimental data processing, it also exhibits good robustness and will not lead to erroneous judgments of other types of irrational numbers due to boundary detection or similar reasons.

### 4.3. The SS of Special Irrational Numbers

For the experiment on special irrational numbers, we selected a total of 200 special irrational numbers to satisfy general conditions. These included decimals, fractions, integers, and negative numbers (if applicable when the root index is odd). For example, $\sqrt[{200}]{\frac{11}{524}}$, $\sqrt[{3253}]{4.12913}$, and $\sqrt[{3}]{-3243.276}$ were demonstrated. Below, we will demonstrate the secret processes of special irrational numbers using $\sqrt[{2514}]{\frac{231}{1453}}$ as an example.

For the root index of 2541, when converted to binary, it is represented as 100111101101 over 12 bits. Following the bit-padding selection principle outlined in the previous text, a 16-bit padding field was determined. The type codes are defined as 0100000100111101101 followed by forty-four random bits and ending with 0, and also as 0110000100111101101 followed by forty-four random bits and ending with 0. Perform the operation as described in Algorithm 2, and thus obtain the secret.

Table 5 is the statistical analysis of our experiment on 200 special irrational numbers.

**Figure 3:**
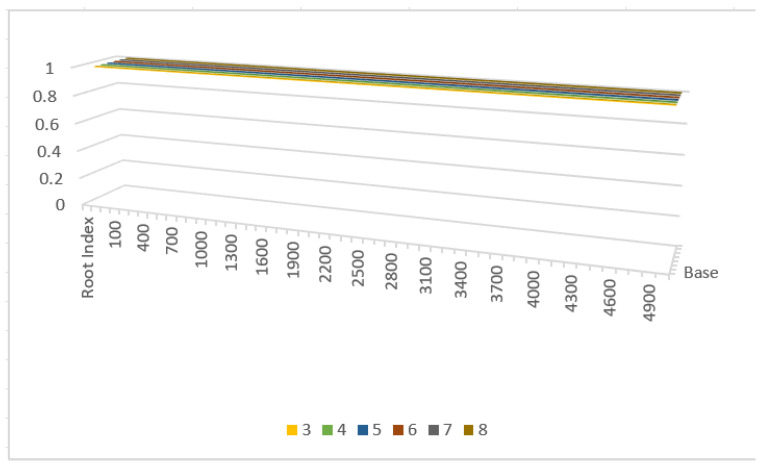
The Impact of Root Index Digit on Experimental Accuracy.

**Figure 4:**
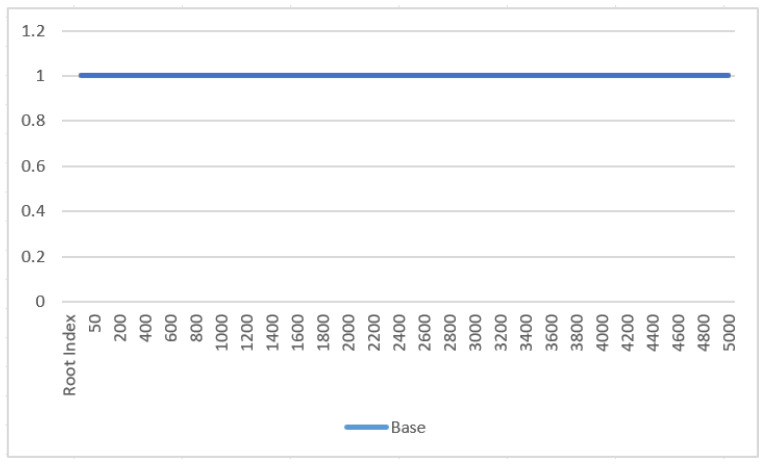
The Impact of Base Digit on Experimental Accuracy.

The Figure 3 and Figure 4 demonstrates the relationship between root index digit size and error rate. From the figure, we can draw the following conclusions:

1. For the protocol special irrational numbers do not suffer from bit loss or errors because the scheme transforms a number that cannot be accurately represented by a computer into an integer usable in the SSS. The receiver can then decode the type code to reveal the transformation used by the sender for the original text;
2. Special irrational numbers are padded with random numbers and allocated reasonable digit lengths, ensuring data security and resistance to attacks. By setting the length of random numbers to more than 50 percent of the total type code length, effective protection against interceptors guessing the raw data is achieved;
3. The type code of special irrational numbers includes fixed features such as the “01” characteristic code, third numerator-denominator mark, and final sign bit, ensuring data integrity.

### 4.4. The SS of Common Irrational Numbers

We selected a total of 28 common irrational numbers, where the numerator is π or *e*, and the denominator ranges from 1 to 20, including both positive and negative values, such as $\frac{\pi }{13}$ and $\frac{-e}{3}$. For example, we will detail the secret sharing scheme for $\frac{\pi }{8}$.

The type for $\frac{\pi }{8}$ is 1101010010001100 and 00110100, where the former indicates that the transmitted value is +π and the latter indicates a positive integer denominator.Perform the operation as described in Algorithm 3, and thus obtain the secret.

Table 6 are the experimental results and error analysis graphs for these 28 data sets:

For common transcendental numbers, there are no errors, as they are known to be transcendental and have been proven to be irrational. We can use specific encodings for π and *e*, which allows the receiver to decode the shared secret without any issues. This encoding method is relatively simple and efficient, with a total length of 16 bits after adding 11 random bits. By using this approach, we maintain both security and efficiency in the protocol.

### 4.5. Comparison with Wang’s Scheme

In Wang’s scheme [30], the formula for converting floating-point numbers into integers is used to implement the SSS:

(7) $$trans\left(x\right)=(x+max(|\theta |))\times 10^{U}$$

The larger the value of *U*, the more decimal places are retained in the parameters, and thus the higher the precision of the reconstructed model parameters. To satisfy the condition that the converted integer range lies within a finite field, *U* must be chosen such that $2*max\left(|\theta |\right)*10^{U}<P$. Furthermore, to maximize *U* while satisfying this condition, the scheme determines the specific value of *U* using the following formula:

(8) $$U=\mathrm{lg}\frac{P}{2max(|\theta |)}$$

We selected 10,000 real numbers of different types, including fractions, finite decimals, recurring decimals, negative numbers, general irrationals, special irrationals, and common irrationals. We performed secret analysis using both Wang’s scheme and our scheme, and tested the sharing accuracy, throughput and transmission delay.

The experimental results are as Table 7. We analyzed the experimental results as follows:

1. Accuracy
  (a) Rational Numbers: Both Wang’s scheme and our scheme achieved 100% accuracy for all subtypes of rational numbers, including fractions, recurring decimals, finite decimals, and negative numbers. This indicates that in handling rational-valued data, both schemes can ensure the integrity and correctness of the data without introducing errors.
  (b) Irrational numbers: Wang’s scheme is unable to handle irrational numbers, as evidenced by the “N/A” entries for common irrationals, special irrationals, and general irrationals. In contrast, our scheme can handle irrational numbers with high accuracy. It achieves 99.9999995% accuracy for common irrationals and 100% accuracy for special and general irrationals. This showcases the broader applicability of our scheme in dealing with a wider range of real-valued data.
2. Throughput
  (a) Rational numbers: For rational numbers, Wang’s scheme generally has a higher throughput than our scheme. For example, for fractions, Wang’s scheme has a throughput of 7329.39 compared to 5663.08 in our scheme. For recurring decimals, the values are 6118.42 and 5337.91, respectively. The only exception is for finite decimals, where our scheme (12,879.99) has a significantly higher throughput than Wang’s scheme (6909.50). This suggests that in most cases of rational number processing, Wang’s scheme can handle data at a faster rate, but our scheme has its own advantage in processing finite decimals.
  (b) Irrational numbers: As Wang’s scheme cannot handle irrational numbers, its throughput for these data types is N/A. Our scheme, however, demonstrates good throughput performance for irrational numbers. It has a throughput of 13,779.41 for common irrationals, 13,471.72 for special irrationals, and 10,075.00 for general irrationals. This highlights that our scheme is capable of efficiently processing irrational-valued data, which is a major advantage in scenarios where such data is present.
    **Table 7:** The comparison with Wang’s scheme.

Data Type		Wang’s Scheme			Our Scheme		
		Accuracy	Throughout	Transmission Delay/µs	Accuracy	Throughout	Transmission Delay/µs
Rational Numbers	Fraction	99.99995%	7329.39	136	100%	5663.08	177
	Recurring Decimals	100%	6118.42	163	100%	5337.91	187
	Finite Decimals	100%	6909.50	145	100%	12,879.99	78
	Negative Numbers	100%	5918.05	169	100%	11,112.58	91
Irrational Numbers	Common Irrationals	N/A	N/A	N/A	99.9999995%	13,779.41	73
	Special Irrationals	N/A	N/A	N/A	100%	13,471.72	74
	General Irrationals	N/A	N/A	N/A	100%	10,075.00	99
3. Transmission Delay
  (a) Rational numbers: For rational numbers, Wang’s scheme has a lower transmission delay in most cases. For fractions, the delay is 136 µs in Wang’s scheme compared to 177 µs in our scheme. For recurring decimals, the delays are 163 µs and 187 µs, respectively. For negative numbers, Wang’s scheme has a delay of 169 µs, while our scheme has 91 µs. The exception is for finite decimals, where our scheme (78 µs) has a lower delay than Wang’s scheme (145 µs). This shows that Wang’s scheme is generally faster in data transmission for rational numbers, except for finite decimals.
  (b) Irrational numbers: Since Wang’s scheme cannot process irrational numbers, its transmission delay for these data types is N/A. Our scheme has a relatively stable transmission delay for irrational numbers, with values of 73 µs for common irrationals, 74 µs for special irrationals, and 99 µs for general irrationals. This indicates that our scheme can handle irrational numbers with a predictable and acceptable level of delay.
4. Overall conclusions
  Wang’s scheme performs well in terms of throughput and transmission delay for most rational numbers, but it lacks the ability to handle irrational numbers. Our Scheme, despite its relatively lower throughput and higher transmission delay for some rational numbers, stands out for its ability to handle both rational and irrational numbers with high accuracy. In applications where data types are diverse and include irrational numbers, such as in scientific computing, advanced financial modeling, and certain healthcare data processing scenarios, our scheme is more suitable. However, in simple rational-number-only applications where speed is a priority, Wang’s scheme may be a better choice. Future improvements to our scheme could focus on optimizing the processing of rational numbers to reduce the performance gap with Wang’s scheme in this aspect while maintaining its strength in handling irrational numbers.

### 4.6. Comparison with the Traditional SSS Scheme

Traditional Shamir secre-sharing (SSS) is inherently designed for integer-based operations over finite fields. In contrast, our proposed scheme extends its capability to real numbers through type encoding.

Table 8 is a detailed comparative analysis based on experimental data.

Performance Dynamics:

1. Traditional SSS achieves lower latency and higher throughput for rational numbers but cannot handle any irrational data types.
2. Our scheme incurs +28.9% latency and −12.4% throughput for rationals but enables 100% coverage of real numbers, including irrationals.

Reasons for Performance Sacrifice:

1. Our scheme embeds type codes in polynomial coefficients (e.g., $a_{1}$ stores the type code), requiring additional computations during share generation and reconstruction. Additionally, rational numbers (e.g., fractions) require both numerator and denominator to be shared as separate integers, doubling the computational load compared to traditional SSS’s single-integer approach.
2. Common irrationals require eight-digit truncation and scaling to integers, adding preprocessing/postprocessing steps absent in traditional SSS; general irrationals such π require reserved bits in type codes, introducing non-trivial encoding/decoding logic.

Impact:

1. In scenarios requiring real-number precision (e.g., financial modeling, quantum systems), our scheme’s applicability outweighs marginal performance losses.
2. Traditional SSS is obsolete in domains where irrational numbers are endemic (e.g., physics, engineering).

The latency and throughput sacrifices in our scheme are deliberate design choices that address a critical gap in traditional SSS: the inability to securely share real numbers. By prioritizing applicability across rational and irrational domains, our approach enables new use cases in fields e.g., financial analytics [31]. While performance optimizations are warranted for specific scenarios, the core trade-off—trading marginal speed for universal numerical support—represents a foundational advancement in secret sharing for the data-driven era.

## 5. Conclusions

The proposed enhanced scheme significantly advances the traditional secret-sharing scheme (SSS) through the innovative incorporation of type code, thereby expanding its functionality and providing more robust system support. Through comprehensive comparative analysis with existing methodologies, our approach demonstrates superior performance metrics in error prevention, standardization, and security enhancement. Empirical evaluations substantiate that the scheme achieves perfect accuracy in the transmission of rational numbers, special irrational numbers, and common irrational numbers, while maintaining negligible error margins for general irrational numbers. Nevertheless, the proposed methodology is not without limitations. The implementation of type coding introduces additional computational overhead, resulting in increased algorithmic complexity and reduced efficiency during the decryption process. Furthermore, while maintaining stringent security requirements, the type code implementation compromises system simplicity, presenting a trade-off that warrants further investigation. Gao et al.’s parallel color-image encryption algorithm—based on a 2D Logistic–Rulkov neuron map—leverages the map’s chaotic dynamics and multi-core parallelism to greatly accelerate large-scale image encryption while enhancing resistance to attacks [32]. The strong anti-interference capability and fast image encryption method in Gao’s scheme have provided significant inspiration for the subsequent optimization of our scheme. We have drawn insights from Gao et al.’s work as guidance for our future scheme improvements.

## Figures and Tables

**Table 1:** The symbol explanations in the scheme.

Symbol	Description
Q	Rational Number
T	Data Type Code
F(x)	Algorithm-generated Share
${\mathrm{CrQ}}_{s}$	Special Irrational Number
${\mathrm{CrQ}}_{p}$	Common Irrational Number
${\mathrm{CrQ}}_{G}$	General Irrational Number
X	Random Bit
s	Positive and Negative Identification Bit
W	The Reserved Bit
U	Precision

**Table 4:** The results of the rational numbers experiment.

Data Types	Quantity	Error Rate
Integer	414	0%
Fraction	449	0%
Decimal	437	0%

**Table 5:** The Results of the Special Irrational Numbers Experiment.

	Base	3	4	5	6	7	8
Root Index							
1000		100%	100%	100%	100%	100%	100%
2000		100%	100%	100%	100%	100%	100%
3000		100%	100%	100%	100%	100%	100%
4000		100%	100%	100%	100%	100%	100%
5000		100%	100%	100%	100%	100%	100%

**Table 6:** The Results of the Common Irrational Number Experiment.

	Common Irrational Numbers	π	e
Divisor			
2–10		100%	100%
10–19		100%	100%
20–29		100%	100%
30–100		100%	100%

**Table 8:** The comparison with traditional SSS Scheme.

Data Type		Traditional SSS Scheme			Our Scheme		
		Accuracy	Throughout	Transmission Delay/µs	Accuracy	Throughout	Transmission Delay/µs
Rational Numbers	Fraction	100%	6466.00	155	100%	5663.08	177
	Recurring Decimals	99.9999995%	6278.74	159	100%	5337.91	187
	Finite Decimals	100%	13,212.92	45	100%	12,879.99	78
	Negative Numbers	100%	18,074.46	55	100%	11,112.58	91
Irrational Numbers	Common Irrationals	N/A	N/A	N/A	99.9999995%	13,779.41	73
	Special Irrationals	N/A	N/A	N/A	100%	13,471.72	74
	General Irrationals	N/A	N/A	N/A	100%	10,075.00	99

## Data Availability

The data presented in this study are available on request from the corresponding author.

## References

[1] Jenifer R.R., Prakash V.S.J. (2024). Rivest-Shamir-Adleman algorithm optimized to protect iot devices from specific attacks. Inform. Autom..

[2] Yang Z., Peng C., Zhong C., Long Y. (2024). Consortium blockchain private key protection scheme based on rational secret sharing and blockchain. Comput. Netw..

[3] Wu C., Zhang L., Xu L., Choo K.K.R., Zhong L. (2024). Privacy-preserving serverless federated learning scheme for Internet of Things. IEEE Internet Things J..

[4] Feng Q., He D., Wang H., Kumar N., Choo K.K.R. (2019). White-box implementation of Shamir’s identity-based signature scheme. IEEE Syst. J..

[5] Bansal G., Sikdar B. (2024). Achieving Secure and Reliable UAV Authentication: A Shamir’s Secret Sharing Based Approach. IEEE Trans. Netw. Sci. Eng..

[6] Shamir A. (1979). How to share a secret. Commun. ACM.

[7] Blakley G.R. (1979). Safeguarding cryptographic keys. Proceedings of the Managing Requirements Knowledge, International Workshop.

[8] Salim S., Suresh S., Gokul R., Reshma S. (2014). Application of Shamir Secret Sharing Scheme for Secret Data Hiding and Authentication. Int. J. Adv. Res. Comput. Sci. Technol..

[9] Kang H., Leng L., Kim B.G. (2022). Data hiding of multicompressed images based on Shamir threshold sharing. Appl. Sci..

[10] Singh P., Raman B. (2018). Reversible data hiding based on Shamir’s secret sharing for color images over cloud. Inf. Sci..

[11] Singh P., Raman B., Misra M. (2018). Just process me, without knowing me: A secure encrypted domain processing based on Shamir secret sharing and POB number system. Multimed. Tools Appl..

[12] Nakkar M., AlTawy R., Youssef A. (2024). Lightweight group authentication scheme Leveraging Shamir’s secret sharing and PUFs. IEEE Trans. Netw. Sci. Eng..

[13] Tejedor-Romero M., Orden D., Marsa-Maestre I., Junquera-Sanchez J., Gimenez-Guzman J.M. (2021). Distributed remote e-voting system based on Shamir’s secret sharing scheme. Electronics.

[14] Yu K., Tan L., Yang C., Choo K.K.R., Bashir A.K., Rodrigues J.J., Sato T. (2021). A blockchain-based shamir’s threshold cryptography scheme for data protection in industrial internet of things settings. IEEE Internet Things J..

[15] Hsu C., Harn L., Xia Z., Zhang M. (2020). Non-interactive dealer-free dynamic threshold secret sharing based on standard shamir’s SS for 5G networks. IEEE Access.

[16] Wang X., Shan H., Yan X., Yu L., Yu Y. (2022). A neural network model secret-sharing scheme with multiple weights for progressive recovery. Mathematics.

[17] Zand A., Orwell J., Pfluegel E. (2020). A secure framework for anti-money-laundering using machine learning and secret sharing. Proceedings of the 2020 International Conference on Cyber Security and Protection of Digital Services (Cyber Security).

[18] Aliasgari M., Blanton M., Bayatbabolghani F. (2017). Secure computation of hidden Markov models and secure floating-point arithmetic in the malicious model. Int. J. Inf. Secur..

[19] Kamm L., Willemson J. (2015). Secure floating point arithmetic and private satellite collision analysis. Int. J. Inf. Secur..

[20] Bogdanov D., Niitsoo M., Toft T., Willemson J. (2012). High-performance secure multi-party computation for data mining applications. Int. J. Inf. Secur..

[21] Bogdanov D., Kamm L., Laur S., Sokk V. (2016). Rmind: A tool for cryptographically secure statistical analysis. IEEE Trans. Dependable Secur. Comput..

[22] Preethi S., Priyadharsini C. (2022). Deep Learning with Blockchain Technology for Secure Data Management in Healthcare Sector using Hybrid Elliptic Curve-Rivest-Shamir-Adleman Cryptography. Cybern. Syst..

[23] Catrina O. (2020). Evaluation of floating-point arithmetic protocols based on shamir secret sharing. Proceedings of the E-Business and Telecommunications: 16th International Conference, ICETE 2019.

[24] Finamore T. (2012). Shamir’s Secret Sharing Scheme Using Floating Point Arithmetic. Ph.D. Thesis.

[25] Gao S., Iu H.H.C., Erkan U., Simsek C., Toktas A., Cao Y., Wu R., Mou J., Li Q., Wang C. (2025). A 3D memristive cubic map with dual discrete memristors: Design, implementation, and application in image encryption. IEEE Trans. Circuits Syst. Video Technol..

[26] Lin Y., Xie Z., Chen T., Cheng X., Wen H. (2024). Image privacy protection scheme based on high-quality reconstruction DCT compression and nonlinear dynamics. Expert Syst. Appl..

[27] Tesoro M., Siloi I., Jaschke D., Magnifico G., Montangero S. (2024). Quantum inspired factorization up to 100-bit RSA number in polynomial time. arXiv.

[28] Isa M., Rahmany N., Asbullah M., Sathar M., Rasedee A. (2019). On the insecurity of generalized (Rivest-Shamir-Adleman)-advance and adaptable cryptosystem. J. Phys. Conf. Ser..

[29] Lemnouar N. (2022). Security limitations of Shamir’s secret sharing. J. Discret. Math. Sci. Cryptogr..

[30] Zhou C., Sun Y., Wang D., Ge Y. (2021). Federal Learning Research Overview. J. Networks Inf. Secur..

[31] Jadhao A.S., Kumbhalkar S.B. (2016). Technical review on secure banking using RSA and AES encryptor methodologies. IOSR J. Electron. Commun. Eng..

[32] Gao S., Zhang Z., Iu H.H.C., Ding S., Mou J., Erkan U., Toktas A., Li Q., Wang C., Cao Y. (2025). A Parallel Color Image Encryption Algorithm Based on a 2D Logistic-Rulkov Neuron Map. IEEE Internet Things J..

